# IFN-γ, TNF-α, IL-10, IL-17 and IL-22 secretion capacities and activation profile of T cells in psoriatic arthritis patients under disease-modifying antirheumatic drug therapy

**DOI:** 10.3389/fimmu.2026.1863954

**Published:** 2026-10-09

**Authors:** Sibel Goksen, Gizem Ayan, Umut Kalyoncu, Gunes Esendagli

**Affiliations:** 1 Department of Medical and Surgical Research, Hacettepe University Institute of Health Sciences, Ankara, Türkiye; 2 Department of Internal Medicine, Division of Rheumatology, Hacettepe University Medical Faculty, Ankara, Türkiye; 3 Department of Basic Oncology, Hacettepe University Cancer Institute, Ankara, Türkiye

**Keywords:** cytokines, disease-modifying antirheumatic drug therapy, immune response, psoriatic arthritis, T cells

## Abstract

**Introduction:**

While biologic disease-modifying antirheumatic drugs (bDMARDs) have transformed management, achieving complete remission remains a challenge due to complex immune networks in Psoriatic arthritis (PsA). This study aimed to characterize the functional modulation of circulating T-helper (Th) and cytotoxic T (Tc) cells in PsA patients following conventional synthetic (csDMARD) and biologic therapy.

**Methods:**

Peripheral blood mononuclear cells were collected from bDMARD-naïve PsA patients at baseline and six months after initiating csDMARD (primarily methotrexate, n=8) or bDMARD (primarily certolizumab, n=11) therapy. Using an optimized flow cytometry-based assay, we evaluated the secretion capacities of TNF-α, IFN-γ, IL-17, IL-22, and IL-10, alongside activation markers (CD40L, ICOS, CD38) in CD4^+^ Th and CD8^+^ Tc cells. Results were associated with clinical disease activity indices (DAPSA, BASDAI, PsAID-12, LEI).

**Results:**

Both therapies significantly improved clinical scores yet failed to achieve immunological quiescence. A central finding was a significant increase in the percentage of TNF-secreting Th and Tc cells post-therapy, which may reflect a compensatory change in T-cell cytokine secretion following treatment. In the bDMARD cohort, Tc cells exhibited a significant expansion of subsets secreting IFN-γ, IL-17, and IL-10, suggesting a possible shift toward Type 1/Type 17-related cytokine responses. Baseline IL-17 and IL-10 levels correlated positively with high disease activity (BASDAI/PsAID-12), while post-treatment IL-17 and IL-22 secretory potential was specifically associated with persistent enthesitis (LEI scores).

**Discussion:**

Despite clinical efficacy, DMARD therapy was associated with persistent alterations in T-cell cytokine profiles, including IFN-γ- and IL-17-producing T-cell populations, particularly within the CD8+ compartment. These findings suggest that peripheral immune profiling may provide additional insights into the immunological effects of therapy and could contribute to future personalized approaches for monitoring immune responses in patients with PsA.

## 1 Introduction

Psoriatic arthritis (PsA) is a complex disease associated with chronic inflammation mainly affecting joints, tendons, entheses, skin, nails, and axial skeleton (1). Neutralization of tumor necrosis factor-α (TNF-α), blockade of interleukin (IL)-17 or IL-12/23, and inhibition of Janus kinases (JAK) are groundbreaking targeted therapy modalities for psoriasis and PsA (2). Nevertheless, heterogeneity amongst the patients and complexity of the immune networks involved in disease pathophysiology are major challenges for a successful treatment of PsA. Randomized studies and real-life data do not indicate significant difference among treatment responses after targeting distinct cytokines and signaling pathways in PsA patients (3). Currently, anti-TNF therapy has been regarded as a favored treatment choice whereas inhibition of IL-17 or IL-12/23 is commonly recommended for the patients with skin involvement (2, 4). Therefore, there is still room for immunologically characterizing the patients who would benefit from a specific choice of these biological disease-modifying antirheumatic drugs (bDMARDs) (2, 5).

Cytokines produced by distinct T cell subsets orchestrate immune responses and have a key role in PsA (4, 6). Especially, interferon (IFN)-γ, tumor necrosis factor (TNF)-α, IL-17, IL-12, IL-22 and IL-23 have been implicated in the PsA-related inflammatory responses. IFN-γ and TNF-α are largely produced by CD4^+^ type-1 helper T (Th1) cells and CD8^+^ cytotoxic T lymphocytes (CTLs) (4, 7). IL-17 and TNF-α are major cytokines secreted by the Th17 subset (8). IL-22 is the signature cytokine of the Th22 cells. IL-12 and IL-23 share a common subunit (p40). IL-12 supports the Th1 differentiation, whereas IL-23 maintains the functional responses by Th17 and Th22 cells (7–9). T cell subsets are widely distinguished according to the cytokine profiles; however, co-expression of various cytokines that are assigned to distinct T cell subsets has also been noted under pathological circumstances (4, 10). Distribution of T cell subsets may be important for determining the prognosis, clinical decision and therapy response in PsA (11).

In this study, upregulation of activation markers (CD40L, ICOS, CD38) and cytokine (IFN-γ, TNF-α, IL-10, IL-17, and IL-22) secretion capacities of helper and cytotoxic T cells obtained from PsA patients under csDMARD or bDMARD therapy were assessed with ex-vivo stimulation analyses. The results were presented in association with clinical findings and therapy responses noted during the follow-up period. Our results indicate the importance of immune activation profile for justification of a treatment decision for PsA.

## 2 Materials and methods

### 2.1 Patients

The patients who were newly diagnosed with psoriatic arthritis (PsA), fulfilled the Classification Criteria for Psoriatic Arthritis (CASPAR), and were biologic disease-modifying antirheumatic drug (bDMARD)-naïve were enrolled in the study. Among the 11 patients who subsequently initiated bDMARD therapy, 4 were receiving conventional synthetic DMARD (csDMARD) treatment at the time of bDMARD initiation, whereas 7 had not received csDMARD treatment for more than 3 months prior to enrollment. Previous csDMARD exposure consisted primarily of methotrexate (MTX; n = 7), while one patient had previously received leflunomide (LEF). Demographic and clinical parameters, mainly as involved domains, were recorded. The presence of axial involvement was primarily based on the physician’s assessment. Disease activity parameters (Disease activity in Psoriatic Arthritis (DAPSA) 66/68, Leeds Enthesisits index (LEI), Body Surface Area (BSA), PASI, Minimal Disease Activity (MDA), the Bath Ankylosing Spondylitis Disease Activity Index (BASDAI score) and outcome measures The Psoriatic Arthritis Impact of Disease 12-item questionnaire (PsAID 12), and Health Assessment Questionnaire (HAQ) scores were recorded (12). DAPSA disease activity categories were defined according to established cut-offs as remission (DAPSA ≤4), low disease activity (DAPSA 5–14), moderate disease activity (DAPSA 15–28), and high disease activity (DAPSA >28). Nineteen patients (n=8 initiated csDMARD, n=11 initiated bDMARD. Patients enrolled to the bDMARD therapy group [female, 72.7%, mean (SD) age, 44.3 (12.8)] had peripheral joint involvement (100%), enthesitis (63.6%), skin involvement (100%), nail involvement (63.6%) and axial disease (9.1%). Patients who were initiated csDMARD [female, 62.5%, mean (SD) age, 45.7 (12.3)] had peripheral joint involvement (100%), enthesitis (25%), skin involvement (87.5%), nail involvement (37.5%). Treatment decisions were certolizumab (10/11) and ustekinumab only for one patient (1/11), according to the individual cytokine profile. The patients who were initiated a bDMARD treatment were reassessed for clinical outcomes, and flow cytometric cytokine secretion assay was done after a 6-month-long follow-up for patients who were initiated a treatment. Demographic and clinical characteristics of patients are summarized in **Table 1**. Blood samples were collected at the initiation and after six months under the therapy. All assessments were made at baseline and six months after the treatment. The study was approved by the Local Ethics Board of Hacettepe University and the Turkish Ministry of Health (KA-21007). In accordance with the Helsinki Declaration, all participants signed a written/informed consent form before the commencement of the study.

**Table 1:** Demographic and clinical characteristics of patients.

Variables	Patients under csDMARD n=8	Patients under bDMARD n=11
**Age,** mean (SD) **Female gender,** n (%) **Education years,** mean (SD) **Smoking** Non-smoker, n (%) Current, n (%) Ex-smoker, n (%) **BMI,** mean (SD) **Psoriasis duration (months),** median (25-75) **Peripheral arthritis,** n (%) **Skin involvement,** n (%) **Enthesitis,** n (%) **Dactylitis,** n (%) **Nail involvement,** n (%) **Axial involvement,** n (%) **SJC (0-66),** mean (SD) **TJC (0-68),** mean (SD) **BSA,** median (25-75) **PASI,** median (25-75) **ESR (mm/hr),** mean (SD) **CRP (mg/L),** mean (SD) **HAQ (0-3),** mean (SD) **BASDAI (0-10),** mean (SD) **VAS PGA (0-10),** mean (SD) **VAS PhGA (0-10),** mean (SD) **PsAID12 (0-10),** mean (SD) **Leeds Enthesitis Index,** median (25-75)	45.7 (12.3) 5 (62.5) 12.4 (4.3) 3 (37.5) 5 (62.5) 0 26.9 (3.5) 150 (48-306) 8 (100) 7 (87.5) 2 (25) 1 (12.5) 3 (37.5) None 1.8 (2.3) 5.4 (3.2) 3 (2-7) 1.5 (0.7-2.2) 10 (7.5) 0.96 (0.99) 0.5 (0.4) 4.8 (2.1) 5.9 (2.6) 5 (2.3) 4.9 (2.1) 0 (0-1.5)	44.3 (12.8) 8 (72.7) 13.5 (3.5) 2 (18.2) 4 (36.4) 5 (45.4) 28.4 (4) 137.8 (36-300) 11 (100) 11 (100) 7 (63.6) None 7 (63.6) 1 (9.1) 1.7 (1.8) 6.7 (9) 4 (3-9) 2.1 (1.1-4.2) 15.6 (13.3) 1.12 (0.6) 0.9 (0.6) 6.9 (2.1) 7.9 (1.8) 6.6 (1.9) 6.9 (1.3) 1 (0-2)

BMI, Body Mass Index; SJC, Swollen Joint Count; TJC, Tender Joint Count; BSA, Body Surface Area; PASI, Psoriasis Area Severity Index; ESR, Erythrocyte Sedimentation Rate; CRP, C-Reactive Protein; HAQ, Health Assessment Questionnaire; BASDAI, The Bath Ankylosing Spondylitis Disease Activity Index; VAS PGA, Visual Analogue Scale Patient Global Assessment; VAS PhGA, Visual Analogue Scale Physician Global Assessment; PsAID12, Psoriatic Arthritis Impact of Disease 12; SD, Standard deviation.Demographic and clinical characteristics are presented as mean (SD), median (25–75), or n (%), as appropriate. Bold text denotes the variable names.

### 2.2 Flow cytometric immunophenotyping and cytokine secretion assay

Peripheral blood mononuclear cells (PBMC) were freshly collected using Ficoll-Paque^®^ (Sigma) 1.077 g/dL density gradient centrifugation. PBMC were washed and resuspended in RPMI-1640 medium supplemented with 10% heat-inactivated fetal bovine serum (FBS), potassium penicillin (100 U/mL) and streptomycin sulphate (100 μg/mL). Otherwise specified, all cell culture reagents were purchased from Biological Industries, USA. T cells were labelled with anti-human-CD3 (SK7), -CD4 (SK3) and -CD8 (RPA-T8), -CD38 (HIT2) and -ICOS (C398.4A) antibodies for testing the T cell activation profiles in unstimulated PBMCs. To induce T cell activation and cytokine secretion, the PBMCs were treated with phorbol-12-myristate-13-acetate (PMA, 20ng/mL) and ionomycin (500 ng/mL) for 6 hours at 37 °C in a humidified 5% CO_2_ incubator. The stimulated PBMC (10^6^ cells) were harvested and washed once in ice-cold PBS (1x) containing 2 mM EDTA and 5% bovine serum albumin (BSA). Following resuspension in RPMI-1640 medium, the cells were labelled with capture antibodies for IFN-γ, TNF-α, IL-10, IL-22, and IL-17 (Miltenyi Biotec) for 5 min on ice, followed by the addition of pre-warmed medium and incubation at 37 °C for 45 minutes. Then, fluorescent-conjugated detection antibodies for the cytokines (Miltenyi Biotec) were added onto the cells along with anti-human-CD3, -CD4, -CD8, and -CD40L (24-31) antibodies. The samples were run on a FACSCanto II flow cytometer (BD Biosciences), and data outputs were analyzed using FlowJo software (version 10.4, TreeStar). Positive cell percentages were determined according to the isotype-matched antibody controls or the autofluorescence values. Representative scatter plots obtained from flow cytometric cytokine secretion assay and immunophenotyping in CD4^+^ helper T cells and CD8^+^ cytotoxic T cells are shown in **Figure 1**. Since PMA stimulation has been reported to induce downregulation and internalization of surface CD4 expression in activated T cells (13), CD4-positive cell populations were interpreted with consideration of this technical limitation.

**Figure 1:**
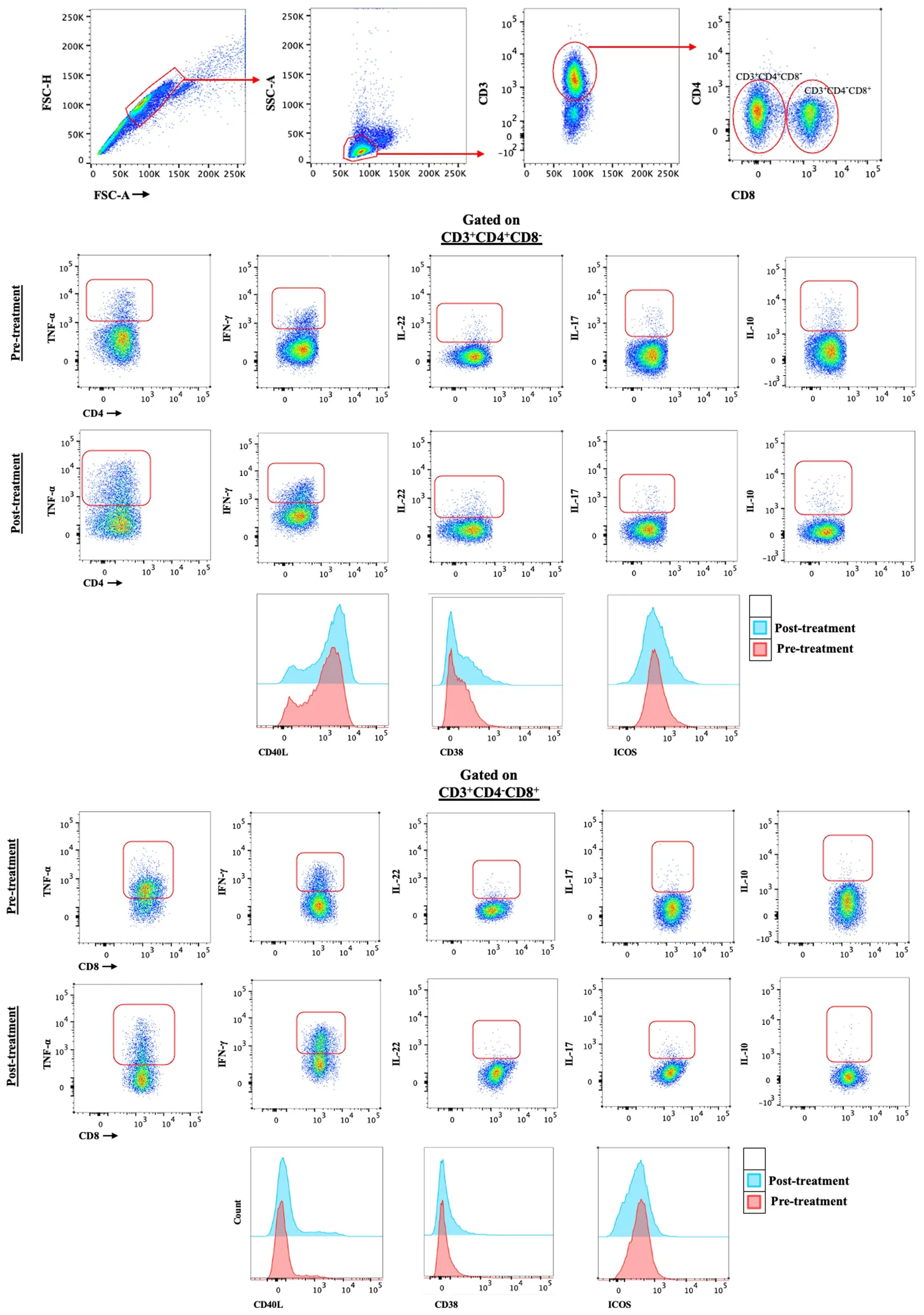
Representative flow cytometry gating strategy for T cell immunophenotyping and cytokine secretion assays. Initially, lymphocytes were identified by gating on singlets, followed by a primary gate based on side scatter (SSC-A) versus forward scatter (FSC-A) properties. Within the lymphocyte gate, T cells were defined as the CD3^+^ population. This population was further subdivided into subsets of interest: CD3^+^CD4^+^CD8^-^ T helper cells and CD3^+^CD4^-^CD8^+^ cytotoxic T cells. The frequency of cytokine-producing cells within these subsets was determined using a flow cytometry-based secretion assay for TNF-α, IFN-γ, IL-17, IL-22, IL-10, CD40L, ICOS, and CD38.

### 2.3 Statistical analysis

Normality was assessed both visually (histograms and probability plots) and analytically (Kolmogorov-Smirnov test, skewness, and kurtosis). Continuous variables were summarized as mean ± standard deviation (SD) or median with 95% confidence intervals (95% CI), as appropriate. Categorical variables were presented as frequencies and percentages. Comparisons between pre-treatment and post-treatment measurements were performed using paired samples t-tests and Wilcoxon signed-rank tests, as appropriate. Given the relatively small sample size and the number of immunological and clinical parameters evaluated, these analyses were considered exploratory. The results were regarded as statistically significant where a “p” value was ≤0.05.

Correlation analysis was performed using the Pearson correlation coefficient to evaluate linear relationships between variables, with coefficients ranging from −1 (strong negative correlation) to +1 (strong positive correlation). All analyses and visualizations were conducted using Python (v3.10). Data processing was carried out using the pandas and numpy libraries, while visualizations were generated with matplotlib and seaborn. These analyses were considered exploratory, and the observed associations were interpreted as hypothesis-generating.

Correlation matrices were displayed in a lower triangular format, employing a blue–white–red color gradient to represent both the direction and magnitude of the correlations. Correlation coefficients were visualized as circles, with circle diameters scaled proportionally to the absolute value of the correlation coefficient (|r|).

A sensitivity analysis was performed by excluding the single patient treated with ustekinumab. Pre- and post-treatment comparisons were then repeated using the same paired statistical tests as in the primary analysis.

## 3 Results

### 3.1 csDMARD therapy modulates the cytokine secretion and activation capacity of helper and cytotoxic T cells in PsA patients

To investigate the effect of conventional synthetic disease-modifying antirheumatic drug (csDMARD) therapy, peripheral blood T-cell subsets were analyzed prior to and six months following the initiation of treatment. Baseline and post-treatment analyses revealed no statistically significant difference in the percentage of CD4^+^ T-helper (Th) cells, CD8^+^ cytotoxic T (Tc) cells, or the CD4^+^/CD8^+^ T-cell ratio (**Figure 2A**). Subsequently, T-cells were stimulated ex-vivo to evaluate their secretory capacity for the cytokines TNF-α, IFN-γ, IL-22, IL-17 and IL-10, as well as the upregulation of CD40L as a functional measure of activation. Consistent with existing literature, TNF- α and IFN- γ were the most frequently secreted cytokines by Th cells in PsA patients. Following csDMARD therapy, the percentage of Th cells positive for the inflammatory cytokines (TNF-α, IFN-γ, IL-17 and IL-22) tended to increase. However, only the increase in the percentage of TNF-α^+^ Th cells achieved statistical significance. The percentage of IL-10-secreting Th cells remained stable and at moderate levels both pre- and post-therapy (**Figure 2B**). Intriguingly, while the percentage of CD40L^+^ Th cells was moderate prior to treatment, it became unequivocally upregulated following csDMARD therapy. This finding may suggest a background immune-modulatory effect induced by the treatment in PsA patients. Additional T-cell activation markers, CD38 and ICOS tested on Th cells, were not significantly modulated by the therapy (**Figure 2B**). Next, the same parameters were assessed in CD8^+^ Tc cells (**Figure 2C**). Similar to the findings in Th cells, the percentage of Tc cells exhibiting TNF-α secretory capacity was significantly increased after csDMARD therapy. Conversely, no significant alterations were observed in the other cytokines or activation-related markers tested in the Tc cell population.

**Figure 2:**
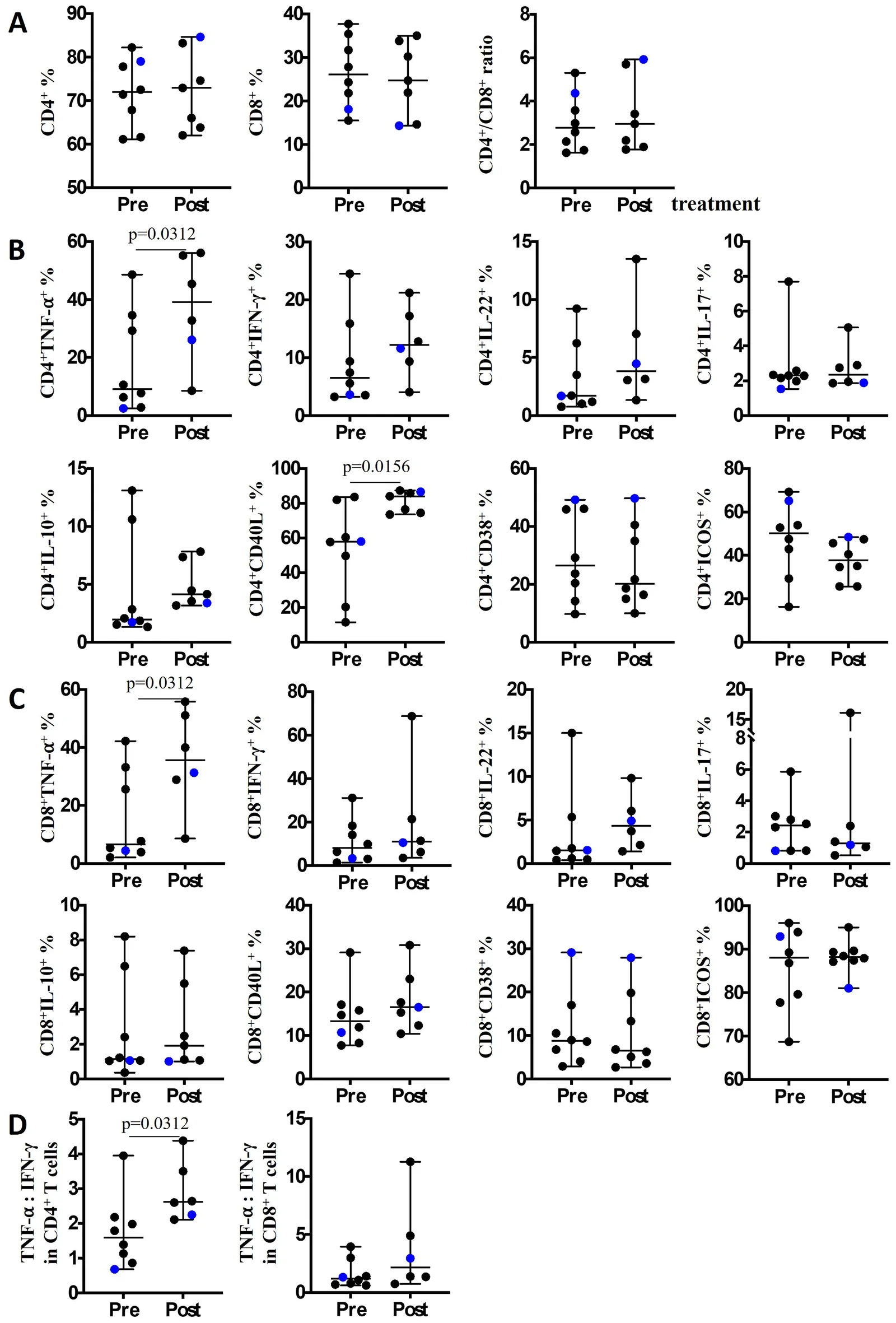
Peripheral blood T cell activation and cytokine secretion profiles before and after csDMARD therapy. A cohort of 8 patients was evaluated at baseline and the 6-month follow-up on csDMARD therapy. PBMCs collected pre- and post-treatment were phenotypically characterized for T cell activation markers (CD3, CD4, CD8, CD38, and ICOS). Following a 6-hour stimulation with PMA and ionomycin, a cytokine secretion assay was employed to quantify the expression of CD40L and the secretion of TNF-α, IFN-γ, IL-17, IL-22, and IL-10. **(A)** Percentage distribution of T cell subsets and corresponding CD4/CD8 ratios. **(B, C)** Percentages of CD4^+^ and CD8^+^ T cells expressing activation markers and secreting cytokines. **(D)** TNF-α^+^:IFN-γ^+^ ratios within CD4^+^ and CD8^+^ T cell subsets. Data are presented as individual scatter dot plots with median values and 95% confidence intervals (95% CI); p < 0.05 was considered statistically significant. The patient receiving a different treatment regimen is highlighted in blue.

Recognizing that TNF-α and IFN-γ could be secreted either simultaneously by the same cell or individually by distinct T cells, a TNF-α:IFN-γ ratio was calculated. The TNF/IFN-γ ratio was evaluated as an exploratory parameter reflecting the relative balance between two major pro-inflammatory T-cell cytokine responses, rather than as a validated biomarker of PsA disease activity or treatment response. This was done to better characterize the impact of csDMARD therapy on the differentiation and activation of Th and Tc cell subsets in PsA patients. In Th cells, the distinction between pre- and post-therapy became more pronounced when analyzed by the TNF-α:IFN-γ ratio (**Figure 2D**). Given that the post-treatment ratio was >1, this suggests that individual Th cells may have acquired an increased capacity for TNF- α production following treatment. In contrast, the change in the TNF-α:IFN-γ ratio for Tc cells did not reach statistical significance.

In summary, the sub-population of both Th and Tc cells demonstrated an elevated potential for TNF-α secretion following csDMARD therapy. Furthermore, Th cells exhibited a notable upregulation of CD40L expression and a tendency toward increased pro-inflammatory capacity.

### 3.2 bDMARD therapy directs the potential of cytotoxic T cells towards IFN-γ and other alternative inflammatory trajectories

Consistent with observations made in the csDMARD cohort, bDMARD therapy did not induce a change in the absolute percentage of circulating Th cells or Tc cells, nor did it alter the CD4^+^/CD8^+^T-cell ratio in the peripheral circulation (**Figure 3A**). Ex-vivo stimulation of Th cells did not reveal marked differences in cytokine secretion profiles between pre- and post-therapy samples. The only exception was that the percentage of Th cells capable of secreting TNF- α was significantly increased following bDMARD therapy, while positivity for all other tested cytokines and activation markers remained unaltered. Of note, the CD40L expression capacity was markedly high in patients both before and after receiving bDMARD therapy (**Figure 3B**). Conversely, a more pronounced change was observed in the Tc cell population. Following bDMARD therapy, the percentage of Tc cells with the capacity to secrete TNF- α, IFN-γ, IL-17, and IL-10 were significantly increased (**Figure 3C**). Specifically, the distribution patterns of TNF-α^+^ Tc cells and IFN-γ^+^ Tc cells were concordant, suggesting a differentiation bias toward an IFN-γ-secreting phenotype in response to bDMARD treatment. No significant differences were found in the expression capacity IL-22, CD38, and ICOS in Tc cells between the pre- and post-therapy cohorts (**Figure 3C**). The calculated TNF-α:IFN-γ ratio was >1 in the Th cell population, indicating a preferential TNF- α response. In the Tc cell population, the ratio remained close to 1. However, no statistically significant difference in this ratio was detected between the pre- and post-treatment groups for either Th or Tc cells (**Figure 3D**).

**Figure 3:**
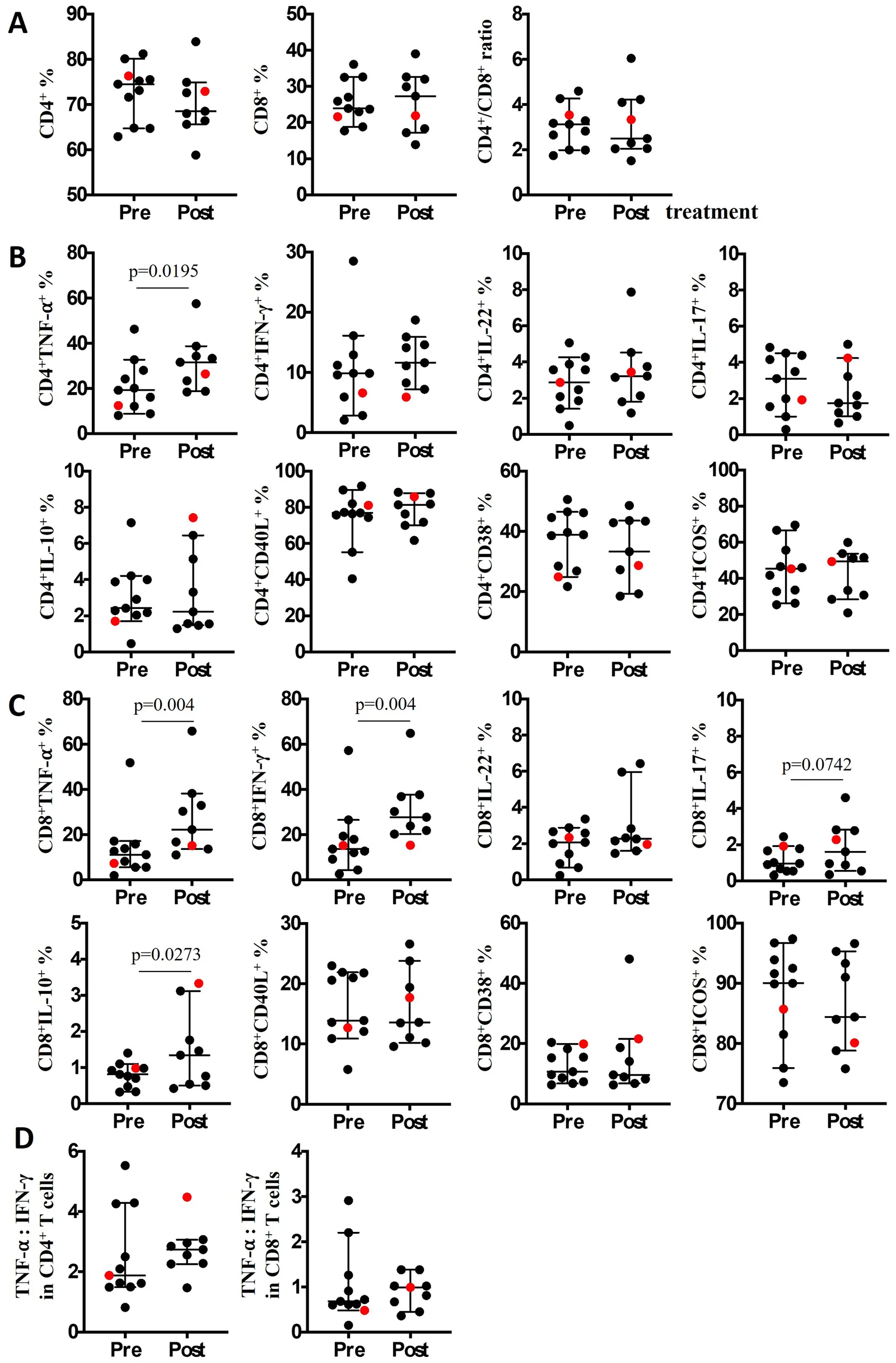
Peripheral blood T cell activation and cytokine secretion profiles before and after bDMARD therapy. A cohort of 11 patients was evaluated at baseline, with 9 patients completing the 6-month follow-up on bDMARD therapy. PBMCs isolated pre- and post-treatment were phenotypically characterized for T cell activation markers (CD3, CD4, CD8, CD38, and ICOS). Following a 6-hour stimulation with PMA and ionomycin, a cytokine secretion assay was utilized to quantify the expression of CD40L and the secretion of TNF-α, IFN-γ, IL-17, IL-22, and IL-10. **(A)** Percentage distribution of T cell subsets and corresponding CD4/CD8 ratios. **(B, C)** Percentages of CD4^+^ and CD8^+^ T cells expressing activation markers and secreting cytokines. **(D)** TNF-α^+^:IFN-γ^+^ ratios within CD4^+^ and CD8^+^ T cell subsets. Data are presented as individual scatter dot plots with median values and 95% confidence intervals (95% CI); p < 0.05 denotes statistical significance. The patient receiving a different treatment regimen is highlighted in red.

Collectively, bDMARD therapy resulted in a modulation of the cytokine secretory profile of Th cells. Simultaneously, the Tc cell population developed a higher potential to contribute to the inflammatory milieu, particularly through the significant increase in multiple pro-inflammatory cytokine-secreting subsets. Although the TNF-α:IFN-γ ratio did not reach significance, the observed value which was compatible in both Th and Tc cells strongly indicates a shift or accentuation of the disease’s inflammatory trajectory toward IFN-γ-mediated pathways following six months of bDMARD treatment.

A sensitivity analysis was performed after excluding the patient treated with ustekinumab. The direction of the changes was preserved for almost all immunological parameters. The increases in CD4^+^ TNF-α, CD8^+^ TNF-α, and CD8^+^ IFN-γ remained statistically significant. CD8^+^ IL-10 showed the same direction of change but did not reach statistical significance after exclusion of this patient, whereas CD8^+^ ICOS became statistically significant. The direction of CD4^+^ ICOS changed, although this parameter was not statistically significant in either analysis (**Supplementary Table 2**).

### 3.3 Association of clinical efficacy of bDMARD therapy and T cell-related immunological parameters in PsA

Following a six-month longitudinal follow-up of patients initiated on bDMARD therapy, significant improvements were observed across primary disease activity indices, including DAPSA, BSA, PASI, and BASDAI; notably, LEI scores remained unchanged. Patient-reported outcomes measured by the PsAID-12 questionnaire were remarkably favorable, whereas HAQ functional scores showed no statistically significant alteration (**Figure 4A**). From a clinical perspective, nearly all patients categorized as having high disease activity (HDA) at baseline transitioned to either low or moderate disease activity following the treatment period (**Figure 4B**). The clinical results were also unchanged after exclusion of the ustekinumab-treated patient. The direction of change was preserved for all clinical parameters, and the significant changes in DAPSA, BASDAI, PASI, VYA, and PsAID-12 remained significant in the sensitivity analysis (**Supplementary Table 2**).

**Figure 4:**
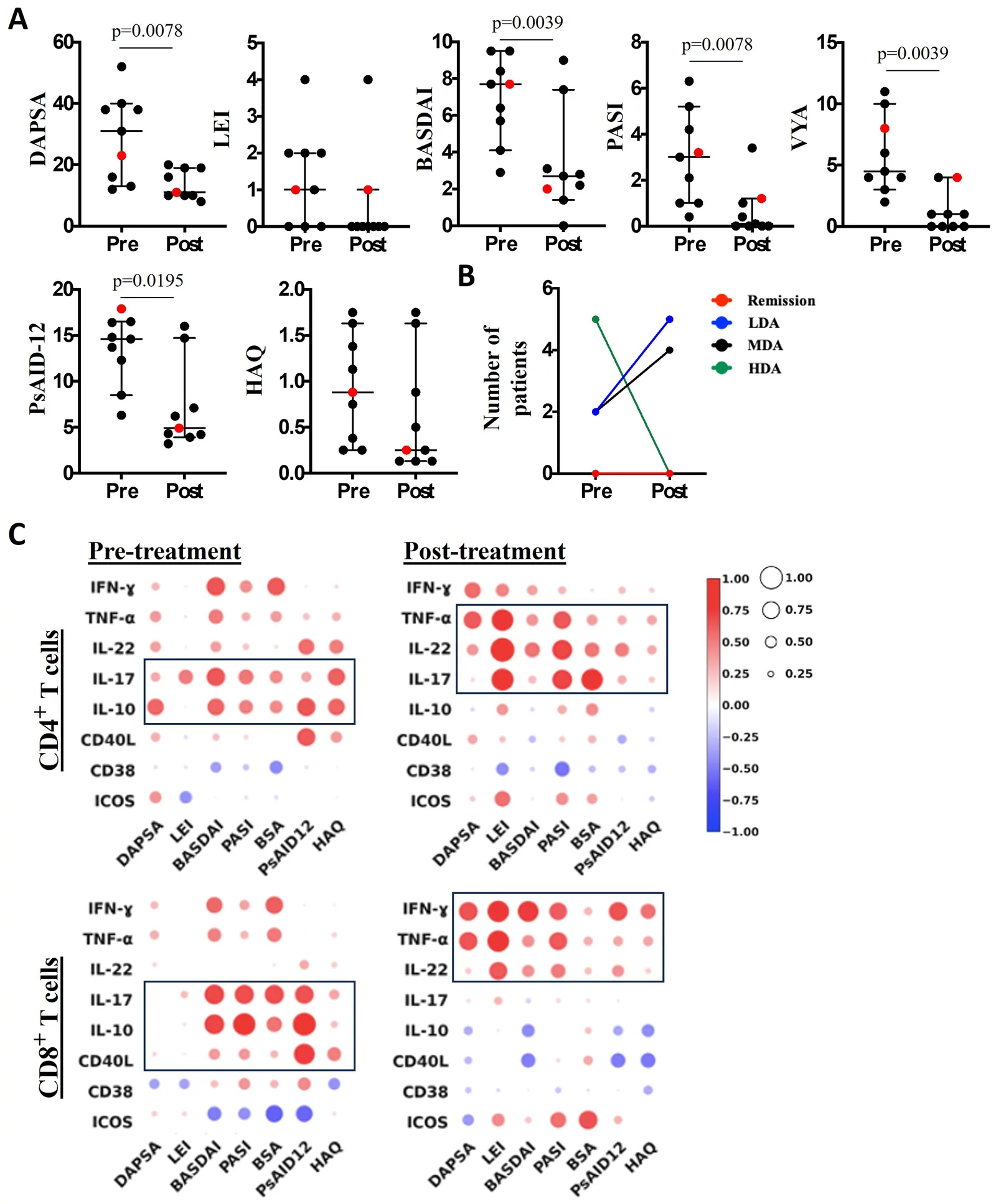
Longitudinal changes in disease activity and their correlation with T cell-related immunological parameters during bDMARD therapy. **(A)** Changes in clinical disease activity scores during the course of bDMARD treatment. **(B)** Distribution of the number of patients according to DAPSA disease activity categories. **(C)** Correlation analysis between disease activity indices and T cell-related immunological parameters. The color scale corresponds to the strength of the correlation, with specific values indicated by the accompanying color gradient key. Size scale depicts the frequency of positivity for each marker. DAPSA, Disease Activity in Psoriatic Arthritis; LEI, Leeds Enthesitis Index; BASDAI, Bath Ankylosing Spondylitis Disease Activity Index; PASI, Psoriasis Area and Severity Index; BSA, Body Surface Area; PsAID-12, Psoriatic Arthritis Impact of Disease 12-item questionnaire; HAQ, Health Assessment Questionnaire. Patients were categorized according to DAPSA disease activity categories: remission (≤4), low (5–14), moderate (15–28), and high (>28). Data are presented as individual scatter dot plots with median values and 95% confidence intervals (95% CI); p < 0.05 denotes statistical significance. The patient receiving a different treatment regimen is highlighted in red.

In the exploratory correlation analysis, baseline (pre-treatment) cytokine secretion and the expression of activation markers by circulating T cells, which were specifically IL-17 and IL-10 for Th cells, and IL-10, IL-17, and CD40L for Tc cells, correlated positively with clinical disease scores. Specifically, BASDAI scores were strongly associated with the Th cell activity, while PsAID-12 scores were linked to the measured Tc cell activities (**Figure 4C**; **Supplementary Table 1**). Post-treatment, the cytokine secretion profiles for both Th and Tc cells shifted. TNF-α, IL-22, and IL-17 became the primary cytokines associated with clinical scores in Th cells, while IFN-γ, TNF-α, and IL-22 emerged as prominent markers in Tc cells. LEI scores demonstrated a correlation with the cytokine secretion capacity across all three cytokines in both Th and Tc cell populations (**Figure 4C**; **Supplementary Table 1**). These findings suggest that bDMARD treatment, primarily certolizumab in this cohort, is associated with changes in the cytokine secretion profile of circulating T cells, including TNF-α, IFN-γ, IL-17, and IL-22. Given the exploratory nature of these analyses, these associations should be interpreted as hypothesis-generating and require confirmation in larger cohorts.

## 4 Discussion

The clinical management of psoriatic arthritis (PsA) has been revolutionized by biologic therapies; however, the underlying immunological shifts that occur during treatment, particularly the response of circulating T-cell compartments, remain complex. Our findings demonstrate that while both csDMARD (primarily methotrexate) and bDMARD (primarily certolizumab) therapies achieve significant clinical efficacy, they induce distinct and somewhat paradoxical modulations of the T-cell cytokine landscape. The heterogeneity of PsA and the absence of standardized immune profiling present significant challenges in biologic (bDMARD) selection (10, 11). We utilized an optimized, flow cytometry-based cytokine secretion assay that offers a rapid (~24h), multiplexed evaluation of T-cell functional phenotypes from minimal peripheral blood volumes. While the local inflammatory microenvironment remains the gold standard for site-specific assessment, peripheral blood profiling provides a minimally invasive and feasible surrogate for estimating systemic immunological status (10, 14). T-cell interactions with myeloid compartments (macrophages, neutrophils, and dendritic cells) drive the PsA inflammatory cascade (10, 15, 16). Our panel comprising TNF-α, IFN-γ, IL-17, IL-10 and IL-22 cytokines was established to delineate the dominant pathogenic pathways within individual patients. The data were further supported by upregulation capacities of T cell activation markers CD40L, ICOS and CD38.

Despite the expansion of the PsA therapeutic armamentarium (e.g., IL-17, IL-12/23, and JAK inhibitors), clinical targets remain unmet; ACR50 and Minimal Disease Activity (MDA) are achieved in only 30-50% and 20-30% of patients, respectively. This disparity highlights the need for better immune profiling for drug selection. Although the study cohort was relatively small, the longitudinal design enabled paired immunological and clinical assessments before and after treatment within the same patients. Furthermore, the bDMARD and csDMARD cohorts were predominantly represented by certolizumab and methotrexate treatment, respectively, providing an opportunity to evaluate treatment-associated changes in well-defined therapeutic settings. These findings support further investigation of immune-profile-guided therapeutic strategies in PsA. Larger randomized controlled studies (RCSs) including a broader range of therapeutic agents and independent validation cohorts are warranted to determine the generalizability of these observations. Future studies should also evaluate the relationship between peripheral cytokine secretion profiles and inflammatory activity at disease sites in order to better define the clinical utility of peripheral immune profiling approaches in PsA.

A central finding in both treatment cohorts was the significant increase in the percentage of T-helper (Th) and cytotoxic T (Tc) cells capable of secreting TNF-α following therapy. In the csDMARD group, this was accompanied by a marked upregulation of CD40L, a critical co-stimulatory molecule for T-cell-driven inflammation. In the bDMARD cohort, which predominantly utilized the TNF-blocker certolizumab, the increase in TNF-α secretory potential was even more pronounced, particularly within the Tc cell population. This phenomenon may represent a “rebound-like” effect or a compensatory biological response. Given that the bDMARD cohort predominantly received the TNF inhibitor certolizumab, sustained or increased TNF-α secretory potential despite clinical improvement may reflect compensatory modulation of peripheral T-cell responses under TNF blockade. However, the present study was not designed to determine whether this increase represents a true biological rebound mechanism. Therefore, these findings suggest that clinical improvement is not necessarily accompanied by complete suppression of the functional cytokine-producing potential of peripheral T cells. Rather, distinct T-cell subsets may remain immunologically active despite effective control of clinical disease activity. In Tc cells, we observed a significant expansion of subsets capable of secreting IFN-γ, IL-17, and IL-10. The concordance between TNF-α and IFN-γ Tc cell patterns suggests a differentiation bias toward a Type 1 (Tc1) cytotoxic phenotype.

This diversification of the inflammatory trajectory is clinically significant. While the primary TNF-mediated pathway is inhibited, the emergence of IL-17, IL-22, and TNF-α as markers positively correlated with post-treatment clinical scores suggests that these cytokines may drive residual disease activity. This is particularly relevant for the LEI (Leeds Enthesitis Index) scores, which remained stubbornly unchanged in our cohort. The correlation between LEI and the increased secretory capacity of IL-17 and IL-22 in T cells supports the hypothesis that enthesitis may be driven by a Th17/Tc17-mediated axis that is less responsive to TNF-inhibition than synovial or cutaneous manifestations. The increased TNF-α secretory potential following both csDMARD and bDMARD therapy may provide a novel perspective on T-cell plasticity and rebound effect following targeted inhibition of cytokine pathways, which aligns with recent literature on “non-responders” or “partial responders”, where T cells exhibit a compensatory up-regulation of pro-inflammatory cytokines when primary pathways are blocked (17). Specifically, the shift toward an IFN-γ and IL-17 “salvage pathway” in CD8^+^ Tc cell population reflects the “Type 1/Type 17” polarization noted in refractory PsA (18). While Th cells are traditionally the focus of PsA research, our findings on Tc cells correlates with literature suggesting that CD8^+^ T cells are also dominant T-cell subsets in the psoriatic plaque and enthesis, often showing a more aggressive inflammatory profile than their CD4^+^ counterparts.

The baseline association between BASDAI and Th-cell activity, contrasted with the association of PsAID-12 with Tc-cell activity, highlights the distinct roles T-cell subsets play in axial versus patient-perceived global disease impact. The post-treatment shift, where PsAID-12 and LEI became more closely associated with Tc-cell cytokine profiles, underscores the underappreciated role of CD8^+^ T cells in the persistence of PsA symptoms. In conclusion, while bDMARD therapy effectively reduces systemic disease activity and transitions patients from HDA to LDA/MDA states, it does not achieve immunological quiescence in the peripheral T-cell compartment. Instead, therapy appears to modulate T-cell potential toward alternative inflammatory trajectories. The observed rebound in TNF-α production capacity and the activation of IFN-γ/IL-17/IL-22 pathways provide a mechanistic rationale for incomplete responses in specific domains like enthesitis and may offer a predictive framework for identifying patients at risk of flare upon treatment discontinuation. However, given the small sample size and the number of correlations examined, these findings should be interpreted as hypothesis-generating and require validation in larger independent cohorts.

TNF-α and IL-17 can be directly targeted and neutralized by specific bDMARDs. IL-12 and IL-23 share a common subunit, p40 (8); therefore, inhibition of IL-12/23 can indirectly reduce the production of IFN-γ in Th1 and cytotoxic T cells by hindering IL-12 (19). On the other hand, IL-23 is necessary for the differentiation of Th17 cells, and its blockade can downregulate IL-17-mediated responses in chronic inflammatory/autoimmune diseases (20–22). As a novel approach, we also considered the impact of IL-22-secreting T cells (as a measure of Th22 cells) in treatment decision. TNF-α and IL-23 are also implicated in the Th22 differentiation (7, 9). Moreover, Th22 cells, which have been implicated in many autoinflammatory and autoimmune diseases, are also capable of producing TNF-α (23). Collectively, a panel of marker cytokines, TNF-α, IFN-γ, IL-17 and IL-22, was established to distinguish different inflammatory pathways involved in PsA. bDMARDs can be readily used for targeting the immune reactions directed by each T cell subset which dominated the patients’ circulating leukocytes. In PsA, IL-10 serves as a critical marker for the homeostatic and regulatory potential of the T-cell compartment. While the percentage of IL-10-secreting Th cells remained stable and at moderate levels following csDMARD therapy, a more dynamic shift was observed in the bDMARD cohort. Specifically, bDMARD treatment, predominantly utilizing TNF-blockers, triggered a significant increase in the percentage of Tc cells capable of secreting IL-10 alongside pro-inflammatory cytokines like IFN-γ and IL-17. This simultaneous upregulation suggests that IL-10 may be part of a broader compensatory feedback loop. The association of elevated baseline IL-10 levels with higher BASDAI and PsAID-12 scores further underscores that, in PsA, IL-10 expression coincides with high disease activity; this points toward a regulatory machinery that is potentially failing to control the underlying pathology.

Despite the clinical relevance of our findings regarding T-cell cytokine modulation in PsA, several limitations must be acknowledged. First, our results are limited by a relatively small, bDMARD-naïve cohort. The size of the study group may restrict the generalizability of the findings to the broader PsA population. Furthermore, within the bDMARD cohort, treatment was primarily restricted to the TNF-blocker certolizumab. Sensitivity analysis excluding the single ustekinumab-treated patient did not substantially change the overall immunological or clinical findings of the bDMARD group. A significant limitation is the absence of a healthy control group or a comparative longitudinal cohort not undergoing DMARD therapy. The study utilized peripheral blood profiling as a minimally invasive surrogate for systemic immunological status. While this approach offers a feasible method for rapid, multiplexed evaluation, the local inflammatory microenvironment (e.g., synovial fluid or skin lesions) remains the gold standard for site-specific assessment. In addition, although the cytokine secretion assay was selected to identify viable cytokine-secreting T-cell subsets at the single-cell level, confirmation of these findings using complementary cytokine detection methods, such as intracellular cytokine staining or ELISA, would further strengthen the interpretation of the results. Finally, while our data suggest that T-cell subtype-based immune profiling could potentially provide additional information relevant to ACR50 and MDA responses alongside current standards of care, larger randomized controlled trials are required. Long-term follow-up is also necessary to determine whether the observed rebound-like T-cell changes are associated with clinical flares upon treatment discontinuation or with the persistence of symptoms in specific domains, such as enthesitis.

In conclusion, this study demonstrates that while both csDMARD and bDMARD therapies significantly improve clinical outcomes in PsA, they are not accompanied by complete immunological quiescence within the peripheral T-cell compartment. Instead, treatment may be associated with a paradoxical modulation of the peripheral T-cell compartment. The increased TNF-α secretory potential, together with the observed changes in IFN-γ- and IL-17-secreting T-cell populations, may be consistent with a rebound-like or compensatory immune response, particularly when considered in the context of previous reports on cytokine-targeted therapy. Although our study was not designed to establish such a mechanism, these findings suggest that clinical improvement may coexist with persistent or altered inflammatory cytokine-producing potential. The associations observed between these T-cell cytokine profiles and residual disease measures, including enthesitis-related scores, should be considered exploratory and hypothesis-generating. Ultimately, these findings support further investigation of multiplexed cytokine profiling as a potential approach for characterizing treatment-associated immune responses in PsA.

## Data Availability

The original contributions presented in the study are included in the article/**Supplementary Material**. Further inquiries can be directed to the corresponding authors.

## References

[1] Gladman DD Antoni C Mease P Clegg DO Nash O . Psoriatic arthritis: Epidemiology, clinical features, course, and outcome. Ann Rheum Dis. (2005) 64:14–7. doi: 10.1136/ard.2004.032482 15708927 PMC1766874

[2] Coates LC Kavanaugh A Mease PJ Soriano ER Acosta-Felquer LM Armstrong AW . Group for research and assessment of psoriasis and psoriatic arthritis 2015 treatment recommendations for psoriatic arthritis. Arthritis Rheumatol. (2016) 68:1060–71. doi: 10.1002/art.39573 26749174

[3] Kerschbaumer A Smolen JS Dougados M De Wit M Primdahl J McInnes I . Pharmacological treatment of psoriatic arthritis: A systematic literature research for the 2019 update of the EULAR recommendations for the management of psoriatic arthritis. Ann Rheum Dis. (2020) 79:S778–86. doi: 10.1136/annrheumdis-2020-217163 32381564

[4] Lowes MA Suárez-Fariñas M Krueger JG . Immunology of psoriasis. Annu Rev Immunol. (2014) 32:227–55. doi: 10.1146/annurev-immunol-032713-120225 24655295 PMC4229247

[5] Ogdie A Coates LC Gladman DD . Treatment guidelines in psoriatic arthritis. Rheumatol (United Kingdom). (2021) 59:I37–46. doi: 10.1093/RHEUMATOLOGY/KEZ383 32159790 PMC7065461

[6] Ezeonyeji A Baldwin H Vukmanovic-Stejic M Ehrenstein MR . CD4 T-Cell dysregulation in psoriatic arthritis reveals a regulatory role for IL-22. Front Immunol. (2017) 8. doi: 10.3389/fimmu.2017.01403 29163483 PMC5666299

[7] Volpe E Servant N Zollinger R Bogiatzi SI Hupé P Barillot E . A critical function for transforming growth factor-β, interleukin 23 and proinflammatory cytokines in driving and modulating human TH-17 responses. Nat Immunol. (2008) 9:650–7. doi: 10.1038/ni.1613 18454150

[8] Schinocca C Rizzo C Fasano S Grasso G La Barbera L Ciccia F . Role of the IL-23/IL-17 pathway in rheumatic diseases: An overview. Front Immunol. (2021) 12. doi: 10.3389/fimmu.2021.637829 33692806 PMC7937623

[9] Benham H Norris P Goodall J Wechalekar MD FitzGerald O Szentpetery A . Th17 and Th22 cells in psoriatic arthritis and psoriasis. Arthritis Res Ther. (2013) 15:R136. doi: 10.1186/ar4317 24286492 PMC3978433

[10] Sakkas LI Bogdanos DP . Are psoriasis and psoriatic arthritis the same disease? The IL-23/IL-17 axis data. Autoimmun Rev. (2017) 16:10–5. doi: 10.1016/j.autrev.2016.09.015 27666819

[11] Miyagawa I Nakayamada S Nakano K Kubo S Iwata S Miyazaki Y . Precision medicine using different biological DMARDs based on characteristic phenotypes of peripheral T helper cells in psoriatic arthritis. Rheumatol (United Kingdom). (2019) 58:336–44. doi: 10.1093/rheumatology/key069 29618121

[12] Mease PJ . Measures of psoriatic arthritis: Tender and swollen joint assessment, psoriasis area and severity index (PASI), nail psoriasis severity index (NAPSI), modified nail psoriasis severity index (mNAPSI), Mander/Newcastle enthesitis index (MEI), Leeds. Arthritis Care Res (Hoboken). (2011) 63:64–85. doi: 10.1002/acr.20577 22588772

[13] Petersen CM Christensen EI Andresen BS Møller BK . Internalization, lysosomal degradation and new synthesis of surface membrane CD4 in phorbol ester-activated T-lymphocytes and U-937 cells. Exp Cell Res. (1992) 201:160–73. doi: 10.1016/0014-4827(92)90360-k 1612121

[14] Dolcino M Ottria A Barbieri A Patuzzo G Tinazzi E Argentino G . Gene expression profiling in peripheral blood cells and synovial membranes of patients with psoriatic arthritis. PloS One. (2015) 10:1–33. doi: 10.1371/journal.pone.0128262 26086874 PMC4473102

[15] Veale DJ Fearon U . The pathogenesis of psoriatic arthritis. Lancet. (2018) 391:2273–84. doi: 10.1016/S0140-6736(18)30830-4 29893226

[16] Skougaard M Ditlev SB Stisen ZR Coates LC Ellegaard K Kristensen LE . Four emerging immune cellular blood phenotypes associated with disease duration and activity established in psoriatic arthritis. Arthritis Res Ther. (2022) 24:1–9. doi: 10.1186/s13075-022-02956-x 36447253 PMC9706839

[17] Skougaard M Søndergaard MF Ditlev SB Kristensen LE . Changes in inflammatory cytokines in responders and non-responders to TNFα inhibitor and IL-17A inhibitor: A study examining psoriatic arthritis patients. Int J Mol Sci. (2024) 25:3002. doi: 10.3390/ijms25053002 38474247 PMC10932211

[18] Steel KJA Srenathan U Ridley M Durham LE Wu S Ryan SE . Polyfunctional, proinflammatory, tissue-resident memory phenotype and function of synovial interleukin-17A+CD8+ T cells in psoriatic arthritis. Arthritis Rheumatol. (2020) 72:435–47. doi: 10.1002/art.41156 31677365 PMC7065207

[19] Langrish CL Chen Y Blumenschein WM Mattson J Basham B Sedgwick JD . IL-23 drives a pathogenic T cell population that induces autoimmune inflammation. J Exp Med. (2005) 201:233–40. doi: 10.1084/jem.20041257 15657292 PMC2212798

[20] Wilson NJ Boniface K Chan JR McKenzie BS Blumenschein WM Mattson JD . Development, cytokine profile and function of human interleukin 17-producing helper T cells. Nat Immunol. (2007) 8:950–7. doi: 10.1038/ni1497 17676044

[21] Qu N Xu M Mizoguchi I Furusawa J Kaneko K Watanabe K . Pivotal roles of T-helper 17-related cytokines, IL-17, IL-22, and IL-23, in inflammatory diseases. Vol 2013. (2013) 2013:968549. doi: 10.1155/2013/968549 23956763 PMC3728507

[22] Batko B . Exploring the diverse immune and genetic landscape of psoriatic arthritis. J Clin Med. (2021) 10:5926. doi: 10.3390/jcm10245926 34945224 PMC8706996

[23] Jiang Q Yang G Xiao F Xie J Wang S Lu L . Role of Th22 cells in the pathogenesis of autoimmune diseases. Front Immunol. (2021) 12. doi: 10.3389/fimmu.2021.688066 34295334 PMC8290841

