# Supplementary material for: IFN-γ, TNF-α, IL-10, IL-17 and IL-22 secretion capacities and activation profile of T cells in psoriatic arthritis patients under disease-modifying antirheumatic drug therapy
Source: Front Immunol. 2026 Oct 9;17:1863954. doi: 10.3389/fimmu.2026.1863954 (PMC13690778; doi:10.3389/fimmu.2026.1863954)
Supplement: Supplementary file 1 [file Table1.docx]

Supplementary Material

# Supplementary Table 1: Correlations between immune parameters and clinical outcomes in patients with psoriatic arthritis (PsA).

|  |  | **Pre-treatment** | | | | | | | **Post-treatment** | | | | | | |
| --- | --- | --- | --- | --- | --- | --- | --- | --- | --- | --- | --- | --- | --- | --- | --- |
|  |  | **DAPSA** | **LEI** | **BASDAI** | **PASI** | **VYA** | **PsAID12** | **HAQ** | **DAPSA** | **LEI** | **BASDAI** | **PASI** | **VYA** | **PsAID12** | **HAQ** |
| **CD4** |  |  | | | | | | |  | | | | | | |
| **IFN-γ** | **r**  **p** | 0,28  0,47 | 0,09  0,82 | 0,65  0,06 | 0,43  0,25 | 0,63  0,07 | 0,10  0,80 | 0,14  0,72 | 0,57  0,11 | 0,46  0,21 | 0,36  0,34 | 0,27  0,48 | -0,11  0,77 | 0,24  0,53 | 0,18  0,64 |
| **TNF-α** | **r**  **p** | 0,39  0,30 | -0,09  0,81 | 0,51  0,16 | 0,30  0,43 | 0,38  0,31 | 0,23  0,55 | 0,30  0,44 | 0,63  0,07 | **0,77**  **0,02** | 0,41  0,28 | 0,64  0,07 | 0,37  0,32 | 0,32  0,41 | 0,34  0,37 |
| **IL-17** | **r**  **p** | 0,32  0,40 | 0,51  0,16 | 0,65  0,06 | 0,52  0,15 | 0,45  0,23 | 0,35  0,36 | 0,61  0,08 | 0,14  0,72 | **0,79**  **0,01** | 0,27  0,49 | **0,71**  **0,03** | **0,77**  **0,02** | 0,31  0,42 | 0,21  0,59 |
| **IL-22** | **r**  **p** | 0,37  0,33 | -0,07  0,87 | 0,37  0,33 | 0,22  0,57 | 0,13  0,75 | 0,56  0,12 | 0,50  0,18 | 0,40  0,29 | **0,85**  **0,004** | 0,53  0,14 | **0,71**  **0,03** | 0,52  0,15 | 0,50  0,17 | 0,32  0,40 |
| **IL-10** | **r**  **p** | 0,58  0,10 | -0,06  0,88 | 0,59  0,09 | 0,49  0,19 | 0,45  0,23 | 0,64  0,06 | 0,59  0,10 | -0,14  0,71 | 0,40  0,29 | -0,14  0,71 | 0,31  0,41 | 0,43  0,24 | -0,02  0,97 | -0,18  0,65 |
| **CD40L** | **r**  **p** | 0,31  0,42 | -0,13  0,74 | 0,24  0,53 | 0,15  0,69 | 0,03  0,94 | 0,63  0,07 | 0,39  0,30 | 0,33  0,38 | 0,22  0,56 | -0,24  0,53 | 0,20  0,61 | 0,28  0,47 | -0,31  0,41 | -0,18  0,64 |
| **CD38** | **r**  **p** | -0,12  0,75 | -0,11  0,77 | -0,37  0,33 | -0,22  0,58 | -0,45  0,22 | 0,13  0,74 | -0,06  0,87 | 0,11  0,77 | -0,44  0,24 | -0,17  0,67 | -0,52  0,15 | -0,24  0,53 | -0,24  0,53 | -0,29  0,44 |
| **ICOS** | **r**  **p** | 0,41  0,27 | -0,42  0,26 | 0,06  0,88 | -0,12  0,76 | -0,13  0,73 | 0,03  0,93 | 0,07  0,87 | 0,19  0,62 | 0,55  0,13 | 0,10  0,80 | 0,43  0,25 | 0,36  0,34 | 0,08  0,84 | -0,19  0,63 |
| **CD8** |  |  | | | | | | |  | | | | | | |
| **IFN-γ** | **r**  **p** | 0,27  0,49 | 0,01  0,99 | 0,57  0,11 | 0,35  0,36 | 0,62  0,08 | 0,09  0,81 | 0,08  0,84 | 0,66  0,05 | **0,77**  **0,02** | **0,74**  **0,02** | 0,62  0,07 | 0,27  0,48 | 0,66  0,05 | 0,53  0,14 |
| **TNF-α** | **r**  **p** | 0,31  0,42 | 0,03  0,94 | 0,48  0,19 | 0,28  0,47 | 0,51  0,16 | 0,03  0,93 | 0,17  0,66 | 0,65  0,06 | **0,77**  **0,01** | 0,43  0,25 | 0,64  0,06 | 0,31  0,42 | 0,34  0,37 | 0,34  0,37 |
| **IL-17** | **r**  **p** | -0,02  0,97 | 0,25  0,53 | **0,70**  **0,04** | **0,68**  **0,04** | **0,68**  **0,04** | 0,66  0,05 | 0,35  0,36 | 0,13  0,74 | 0,28  0,47 | -0,16  0,67 | 0,15  0,70 | 0,14  0,72 | -0,09  0,82 | -0,02  0,96 |
| **IL-22** | **r**  **p** | 0,15  0,70 | -0,03  0,94 | -0,04  0,93 | -0,04  0,92 | -0,10  0,81 | 0,32  0,40 | 0,20  0,62 | 0,20  0,60 | 0,64  0,06 | 0,42  0,25 | 0,51  0,16 | 0,24  0,53 | 0,42  0,27 | 0,19  0,63 |
| **IL-10** | **r**  **p** | -0,05  0,91 | -0,12  0,75 | **0,71**  **0,03** | **0,79**  **0,01** | 0,54  0,14 | **0,81**  **0,008** | 0,24  0,54 | -0,31  0,42 | 0,10  0,81 | -0,45  0,20 | 0,03  0,93 | 0,23  0,55 | -0,34  0,37 | -0,44  0,23 |
| **CD40L** | **r**  **p** | 0,15  0,69 | 0,10  0,80 | 0,39  0,30 | 0,40  0,29 | 0,27  0,49 | **0,74**  **0,02** | 0,49  0,18 | -0,26  0,49 | -0,003  0,99 | -0,50  0,17 | 0,14  0,71 | 0,33  0,39 | -0,51  0,16 | -0,52  0,15 |
| **CD38** | **r**  **p** | -0,35  0,35 | -0,36  0,35 | 0,25  0,52 | 0,40  0,28 | 0,28  0,47 | 0,45  0,22 | -0,41  0,27 | -0,21  0,58 | -0,13  0,73 | -0,07  0,86 | -0,21  0,59 | -0,11  0,77 | -0,08  0,84 | -0,31  0,41 |
| **ICOS** | **r**  **p** | 0,19  0,63 | 0,16  0,68 | -0,50  0,18 | -0,43  0,25 | -0,60  0,09 | -0,58  0,10 | 0,13  0,75 | -0,39  0,30 | 0,45  0,22 | 0,22  0,57 | 0,55  0,12 | 0,66  0,05 | 0,28  0,46 | 0,01  0,97 |

Correlation analyses were performed using Pearson correlation coefficients. Correlation coefficients (r) and corresponding p-values are presented for associations between cytokine-secreting T-cell populations, T-cell activation markers, and clinical outcome measures. Statistically significant correlations (p ≤ 0.05) are highlighted in bold.

**Supplementary Table 2.** Sensitivity analysis of bDMARD-associated immunological and clinical changes after exclusion of the ustekinumab-treated participant

|  |  | Primary analysis | Sensitivity analysis | Direction of change |
| --- | --- | --- | --- | --- |
| A. ***Immunological parameters*** | | | | |
| **CD4** | **TNF-⍺** | 0.0195 (*) | 0.0391 (*) | Preserved |
|  | **IFN-γ** | 0.5703 | 0.5469 | Preserved |
|  | **IL-22** | 0.4258 | 0.5469 | Preserved |
|  | **IL-17** | 0.8203 | 0.4609 | Preserved |
|  | **IL-10** | 0.4258 | 0.7422 | Preserved |
|  | **CD40L** | 0.6523 | 0.8438 | Preserved |
|  | **CD38** | 0.4766 | 0.2344 | Preserved |
|  | **ICOS** | 0.6523 | 0.3125 | Changed |
| **CD8** | **TNF-⍺** | 0.0039 (**) | 0.0078 (**) | Preserved |
|  | **IFN-γ** | 0.0039 (**) | 0.0078 (**) | Preserved |
|  | **IL-22** | 0.2031 | 0.1484 | Preserved |
|  | **IL-17** | 0.0742 | 0.1094 | Preserved |
|  | **IL-10** | 0.0273 (*) | 0.0547 | Preserved |
|  | **CD40L** | 0.8203 | 0.9453 | Preserved |
|  | **CD38** | 0.3594 | 0.5469 | Preserved |
|  | **ICOS** | 0.2031 | 0.0391 (*) | Preserved |
| **CD4** |  | 0.3594 | 0.6406 | Preserved |
| **CD8** |  | 0.5703 | 0.5469 | Preserved |
| **CD4/CD8 ratio** |  | 0.5703 | 0.4609 | Preserved |
| **TNF-⍺ : IFN-γ in CD4^+^ T cells** | | 0.6523 | 0.9453 | Preserved |
| **TNF-⍺ : IFN-γ in CD8^+^ T cells** | | 0.4961 | 0.7422 | Preserved |
| B. ***Clinical parameters*** | | | | |
| **DAPSA** |  | 0.0078 (**) | 0.0156(*) | Preserved |
| **LEI** |  | 0.3125 | 0.3125 | Preserved |
| **BASDAI** |  | 0.0039 (**) | 0.0078 (**) | Preserved |
| **PASI** |  | 0.0078 (**) | 0.0156 (*) | Preserved |
| **VYA** |  | 0.0039 (**) | 0.0078 (**) | Preserved |
| **PSAID12** |  | 0.0195 (*) | 0.0391 (*) | Preserved |
| **HAQ** |  | 0.0859 | 0.1719 | Preserved |

A post hoc sensitivity analysis was performed after exclusion of the single ustekinumab-treated participant, resulting in a certolizumab-only cohort of 10 patients. Pre- and post-treatment comparisons of immunological and clinical parameters were repeated using the same paired statistical tests as in the primary analysis. “Preserved” indicates that the direction of the pre- to post-treatment change remained consistent with the primary analysis, whereas “Changed” indicates a reversal in direction. Nominal statistical significance was defined as P<0.05. *P<0.05; **P<0.01.
